# Theoretical Investigations on the Sensing Mechanism of Phenanthroimidazole Fluorescent Probes for the Detection of Selenocysteine

**DOI:** 10.3390/molecules27238444

**Published:** 2022-12-02

**Authors:** Zhe Tang, Xiaochen Wang, Runze Liu, Panwang Zhou

**Affiliations:** 1 Tianjin Key Laboratory of Drug Targeting and Bioimaging, Life and Health Intelligent Research Institute, Tianjin University of Technology, Tianjin 300384, China; 2 Institute of Molecular Sciences and Engineering, Institute of Frontier and Interdisciplinary Science, Shandong University, Qingdao 266237, China; 3 Key Laboratory of Materials Modification by Laser, Ion and Electron Beams, Ministry of Education, Dalian University of Technology, Dalian 116024, China

**Keywords:** selenocysteine, ESIPT, PET, frontier molecular orbital

## Abstract

The level of selenocysteine (Sec) in the human body is closely related to a variety of pathophysiological states, so it is important to study its fluorescence sensing mechanism for designing efficient fluorescent probes. Herein, we used time-dependent density functional theory to investigate the fluorescence sensing mechanism of phenanthroimidazole derivates A4 and B4 for the detection of Sec, which are proposed to be designed based on excited state intramolecular proton transfer (ESIPT) and intramolecular charge transfer (ICT) mechanisms. The calculation results show that the fluorescence quenching mechanism of A4 and B4 is due to the photo-induced electron transfer (PET) process with the sulfonate group acts as the electron acceptor. Subsequently, A4 and B4 react with Sec, the sulfonate group is substituted by hydroxyl groups, PET is turned off, and significant fluorescence enhancement of the formed A3 and B3 is observed. The theoretical results suggest that the fluorescence enhancement mechanism of B3 is not based on ICT mechanism, and the charge transfer phenomenon was not observed by calculating the frontier molecular orbitals, and proved to be a local excitation mode. The reason for the fluorescence enhancement of A3 based on ESIPT is also explained by the calculated potential energy curves.

## 1. Introduction

Selenocysteine (Sec) is an important member of the reactive selenium species and an important component of selenoprotein (SeP) [1] because the content of Sec is closely related to the catalytic ability of SeP, which further affects a variety of pathological and physiological states in the human body, such as cancer, diabetes, neurodegenerative diseases, cardiovascular disease, and male infertility [2,3,4]. Therefore, how to detect Sec quickly, efficiently, and accurately is a huge challenge faced by researchers. Compared with high-performance liquid chromatography, electrochemical assay, thin layer chromatography, mass spectrometry, capillary electrophoresis, titration analysis, Fourier-transform infrared spectroscopy, ultraviolet-visible spectroscopy [5,6,7,8,9,10,11], fluorescence detection technology can better protect the integrity of biological samples and has the advantages of fast response, low cost, simplicity, high sensitivity, huge potential, etc. [12]. That is to say, researchers need to develop fluorescent probes with high biocompatibility and selectivity to monitor Sec levels.

Excited state intramolecular proton transfer (ESIPT) reaction is one of the important fundamental reactions in photochemistry and photo physical, which is a four-level cyclic process and usually exhibits dual fluorescence characteristic from the normal form N* (or Enol*) and the tautomeric form T* (or Keto*) due to the proton transfer [13,14,15,16,17]. Based on this characteristic, the ESIPT system has made great contributions in the fields of UV filters, laser dyes, white light emitting materials, molecular switches, fluorescent probes, organic optoelectronic materials, etc. [18,19,20,21,22,23,24]. In particular, fluorescent probes developed based on the ESIPT system, also known as ESIPT-based fluorescent probes, can minimize self-absorption and reduce interference from auto-fluorescence due to their in vivo application [25,26]. Therefore, more and more scientists are designing new ESIPT-based fluorescent probes to detect Sec levels, and they are proving to have good application prospects. In addition, intramolecular charge transfer (ICT) processes also play an important role in the field of fluorescent probes because of their special photo physical properties [27]. Upon photo-excitation, ICT-based fluorescent probes typically occur on an ultrafast time scale and produce a large Stokes shift in the emission spectrum.

**Scheme 1:**
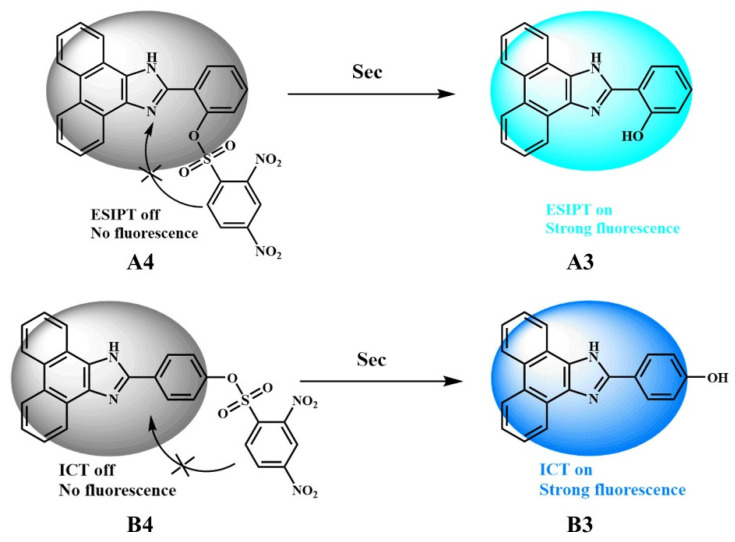
Mechanism of Sec Sensing by A4 and B4.

Recently, Wang et al. synthesized two phenanthroimidazole turn-on probes, A4 and B4 (isomers), as turn-on fluorescent probes for the detection of Sec [28]. They found that A4 and B4 have high selectivity and sensitivity to Sec and can be applied to fluorescence imaging of Sec in living cells. Among them, A4 and B4 were designed according to the ESIPT and ICT mechanisms, respectively. In other words, when A4 and B4 react with Sec, the sulfonate group is replaced by the hydroxyl group, photo-induced electron transfer (PET) is turned off, and significant fluorescence enhancement of the formed A3 and B3 is observed due to the turned-on ESIPT and ICT mechanisms (Scheme 1). It is worth mentioning that the PET from donor (D) to acceptor (A) leads to the formation of a charge separated state composed of donor free radical cations and acceptor free radical anions. (1,2) In the intramolecular PET process, D and A coexist in the same molecule, while in the intermolecular PET process, they correspond to different molecules. Generally, PET responsible for fluorescence quenching [29]. However, we found that Wang et al. did not provide a detailed explanation for the fluorescence quenching of A4 and B4, and also overlook the enhanced fluorescence of A3 after the occurrence of ESIPT [28]. Therefore, a detailed theoretical investigation of the fluorescence quenching mechanism of A4 and B4 and the fluorescence enhancement mechanism of A3 and B3 is necessary.

In this work, we use the time-dependent density functional theory (TD-DFT) method to reveal the fluorescence quenching mechanism of A4 and B4 and the fluorescence enhancement mechanism of A3 and B3. The calculated electronic excitation energies, corresponding oscillator intensities, corresponding compositions, and frontier molecular orbitals (FMOs) well reveal that the fluorescence quenching of A4 and B4 is due to the PET mechanism, and the sulfonate group was found to be the main cause of PET formation. Meanwhile, the calculated energy and potential energy curves indicate that the forward occurrence of the ESIPT process in A3 is easier and more structurally stable than the reverse, which is responsible for the enhanced fluorescence after the occurrence of ESIPT in A3. In particular, according to the analysis of FMOs and Δr index, it was confirmed that B3 did not undergo ICT processes, but only normal local excitation characteristics. Thus, we provide a reasonable sensing mechanism that will help scientists develop new fluorescent probes for the detection of Sec.

## 2. Theoretical Methods

In this paper, all theoretical calculations are carried out in Gaussian 16 [30]. The ground state (S_0_) and the first excited state (S_1_) of A3, A4, B3, and B4 are optimized by using the B3LYP functional and TZVP basis set in DFT and TDDFT methods, respectively [31,32,33,34,35]. For considering dispersion force, all the simulations have been performed by adopting the D3 version of Grimme’s dispersion [36]. Exploring the effects brought by surrounding environments, this work adopts the integral equation formal variables of the polarizable continuum model (IEFPCM) model for all simulations [37,38,39], using water (ε = 78.3553) solvent. By further checking the stability of all structures via infrared (IR) vibrational analysis, we confirmed that all optimized geometries are local minima without imaginary frequency. In addition, the calculated electron data and FMOs are the same as those of optimization and are used for the study of fluorescence properties. To interpret the ESIPT mechanism by the PECs, we construct the PECs of S_0_ and S_1_ states along with the forward direction of the reaction path. In particular, the PECs construction is relaxation scanning. Importantly, since the CAM-B3LYP functional can better describe charge transfer [40], we also used the CAM-B3LYP-D3/TZVP/IEFPCM level to calculate the excitation and emission energies and their related oscillator strengths, components and FMOs. We also provide some important data in the supporting information (Tables S1–S4, Figures S1 and S2, and all the optimized geometries).

## 3. Results and Discussion

### 3.1. A3 and A4

**Figure 1:**
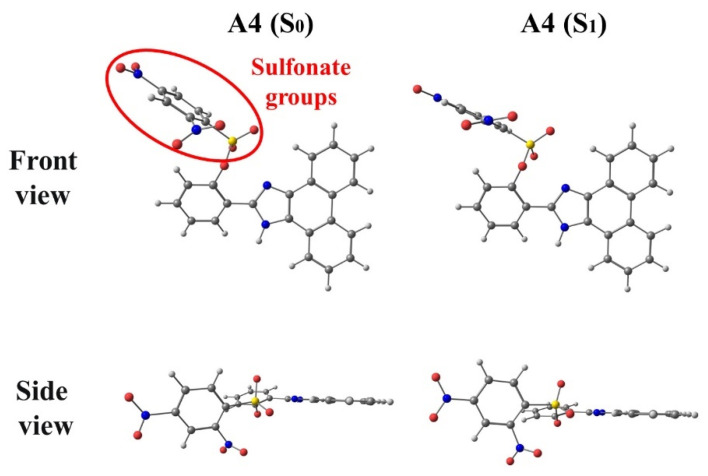
B3LYP-D3/TZVP/IEFPCM-optimized geometries of A4 in the S_0_ and S_1_ state.

**Figure 2:**
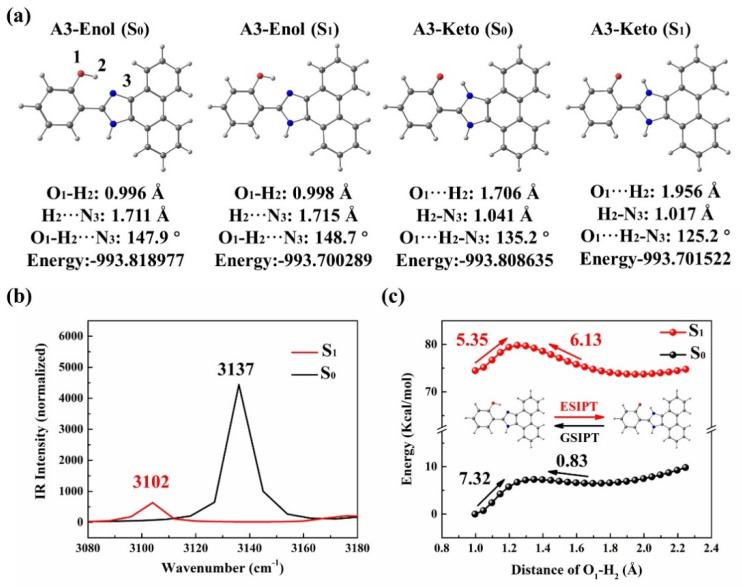
B3LYP−D3/TZVP/IEFPCM-optimized geometries of A3−Enol and A3−Keto in the S_0_ state (**a**); IR vibrational spectra of A3 involved in O_1_−H_2_ stretching modes in S_0_ and S_1_ states (**b**); Potential energy curves of the S_0_ and S_1_ states for A3 along with O_1_−H_2_···N_3_ (**c**).

In Figure 1, we provide the front and side views of the A4 geometry in the S_0_ and S_1_ states, respectively. It can be seen that the change of the sulfonate groups in A4 is more obvious, and we assume that this is caused by the strong electron absorption ability of this group. Here, we suspect that the cause of A4 fluorescence quenching may be caused by the sulfonate group, which we also verify in the later calculation content. To better compare with the A4 geometry, we also calculated the A3 geometry, and since the ESIPT mechanism is involved, we provide the A3−Enol and A3−Keto in the S_0_ and S_1_ states, respectively (Figure 2a). We have labeled the most important atoms in intramolecular hydrogen bonds with numbers 1–3 for better understanding. It can be seen that the A3 geometry does not change significantly in the S_0_ and S_1_ states, because the hydroxyl group replaces the sulfonic group and the structural rigidity is enhanced. The primary bond parameters (lengths and angles) of the A3−Enol and A3−Keto forms in water solvents are shown in Figure 2a. Calculation of A3−Enol geometric parameters shows that the O_1_−H_2_ bond length increases from 0.996 Å (S_0_ state) to 0.998 Å (S_1_ state) and the δ (O_1_−H_2_∙∙∙O_3_) bond angle expands from 147.9° to 148.7°, indicating that the intramolecular hydrogen bonding is enhanced in the S_1_ state. In particular, we found that the H_2_−N_3_ bond length increased in the S_1_ state, which would be detrimental to the occurrence of ESIPT.

To further prove our idea, we calculated the IR vibrational spectra of A3−Enol forms for the analysis of the intramolecular hydrogen bond (Figure 2b). For A3−Enol forms in a water solvent, the calculated O_1_−H_2_ stretching vibrational frequency of the A3−Enol forms was changed from 3137 cm^−1^ (S_0_ state)→3102 cm^−1^ (S_1_ state). We can see that the O_1_−H_2_ stretching modes of the 2P3HBQ−Enol and 2HP3HBQ−Enol forms both exhibit a redshift of 25 cm^−1^, indicating enhanced intramolecular hydrogen bonding in the S_1_ state. Although the IR vibration spectrum shows that the redshift is in the S_1_ state, the change in the redshift value is relatively small, so it is also easy to judge whether the ESIPT process occurs. To more intuitively judge whether the ESIPT process occurs, we constructed the PECs of A3 in the S_0_ and S_1_ state, with a function of O_1_−H_2_ bond length in the step of 0.05 Å (Figure 2c). For A3 fluorophore, the energy potential barrier of A3−Enol→A3−Keto is 7.32 kcal/mol in the S_0_ state and 5.35 kcal/mol in the S_1_ state. The energy potential barrier of A3−Keto→A3−Enol is 0.83 kcal/mol in the S_0_ state and 6.13 kcal/mol in the S_1_ state. These results show that Keto→Enol is more likely to occur in the S_0_ state, and Enol→Keto is more likely to occur in the S_1_ state. In addition, we can see that A3−Keto is more stable in the S_1_ state than A3−Enol (Figure 2a). These results directly prove the main reason for A3−Keto* emission and reasonably explain the experimental phenomenon.

To understand the excitation and emission processes of A3 and A4, we calculated the electronic excitation energy and emission energy (Table 1 and Table 2), as well as the corresponding oscillator intensities and compositions. It can be seen that the S_0_→S_1_ transition is allowed for A3, so the oscillator intensity is 0.5677, which indicates that the S_1_ state is a bright state. The calculated electron excitation energy is 3.58 eV (Table 1), which is consistent with the experimental results [28]. In addition, the calculated emission energies of A3−Enol and A3−Keto are 2.99 eV and 2.76 eV (Table 2), respectively, which are also in good agreement with the experimental results [28], indicating that our calculation method is reasonable. However, it can be seen that the S_0_→S_1_ transition is forbidden for A4, so the oscillator intensity is 0.0013. We find that the S_0_→S_7_ transition is allowed because the oscillator intensity of the S_0_→S_7_ transition is 0.6518. Meanwhile, the calculated emission energy is 0.98 eV, and the oscillator intensity is 0, so the S_1_→S_0_ transition undergoes a non-radiative pathway.

**Figure 3:**
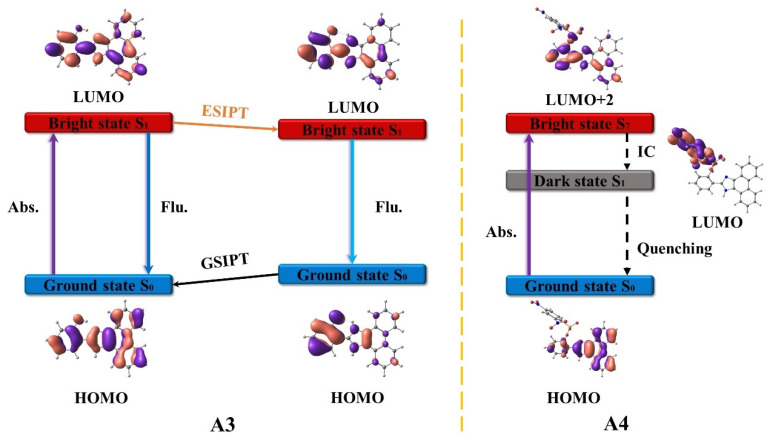
Scheme of the different sensing mechanisms and relevant frontier molecular orbitals A3 and A4.

Based on the above results, we added FMOs analysis to further elaborate the sensing mechanism of A3 and A4 (Figure 3). The calculation results show that the S_0_→S_1_ transition of A3 is mainly H→L, and the S_1_→S_0_ transition is mainly L→H, so we provide HOMO and LUMO. It can be seen that H→L is ππ * features, which provides evidence that the S_1_ state is a bright state. Therefore, the sensing mechanism of A3 can be summarized: Firstly, the A3 excitation transitions to the S_1_ state, then vibrationally relaxes to a stable A3−Enol* in the S_1_ state. One part of the energy emits fluorescence back to the S_0_ state (A3−Enol), the other part of the energy overcomes the energy barrier and stabilizes to A3−Keto* through the ESIPT process, then emits fluorescence back to the S_0_ state (A3−Keto), and finally returns to stable A3-Enol. Besides, the S_0_→S_1_ and S_0_→S_7_ transition of A4 are mainly H→L and H→L+2, respectively, and the S_1_→S_0_ transition is mainly L→H, so we provide HOMO, LUMO, and LUMO+2. In Figure 3, it can be seen that the H→L+2 is ππ * features with the ICT process. However, the H→L shows a classic PET process. It is well known that the PET process is a non-radiative transition process, which is also the main reason for A4 fluorescence quenching. Therefore, the sensing mechanism of A4 can be summarized: After photoexcitation, A4 transitions from the S_0_ state to the S_7_ state and then undergoes an ultrafast internal conversion to reach the S_1_ state accompanied by the PET, and nonradiative return to the S_0_ state.

### 3.2. B3 and B4

**Figure 4:**
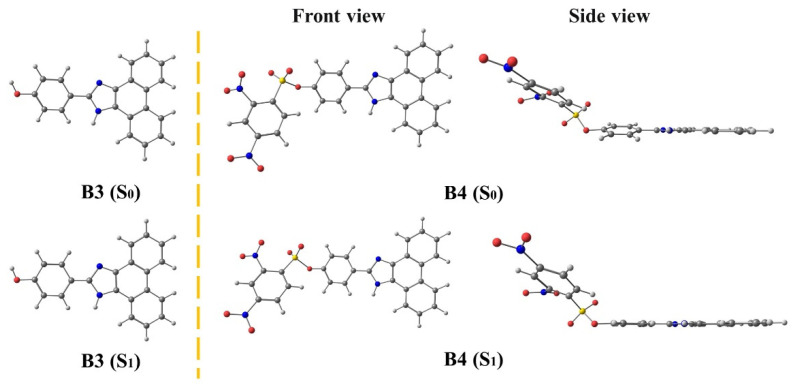
B3LYP−D3/TZVP/IEFPCM-optimized geometries of B3 and B4 in the S_0_ and S_1_ state.

In Figure 4, the B3 and B4 geometries are also optimized in the S_0_ and S_1_ states. The calculation results show that the geometry of B3 does not change significantly in the S_0_ and S_1_ states, while the geometry of B4 changes significantly in the S_0_ and S_1_ states, which is also due to the sulfonate group. Therefore, we believe that the fluorescence quenching mechanism of B4 is also related to the sulfonate group. In order to prove our point of view, the excitation and emission energies, as well as their related oscillator intensities and compositions are also calculated (Table 3 and Table 4). The S_0_→S_1_ transition is allowed for B3, so the oscillator intensity is 0.2500, which indicates that the S_1_ state is a bright state. The calculated electron excitation energy is 3.57 eV (Table 3), which is consistent with the experimental results [28]. In addition, the calculated emission energies of B3 is 2.81 eV (Table 4), which are also in good agreement with the experimental results [28]. Similarly, it can be seen that the S_0_→S_1_ transition is forbidden for A4, so the oscillator intensity is 0.0085, which indicates that the S_1_ state is a dark state. We also found that the S_7_ state is a bright state, because the oscillator intensity of the S_0_→S_7_ transition is 0.5826. Meanwhile, the calculated emission energy is 0.97 eV, and the oscillator intensity is 0.0243, so the S_1_→S_0_ transition undergoes a non-radiative pathway.

**Figure 5:**
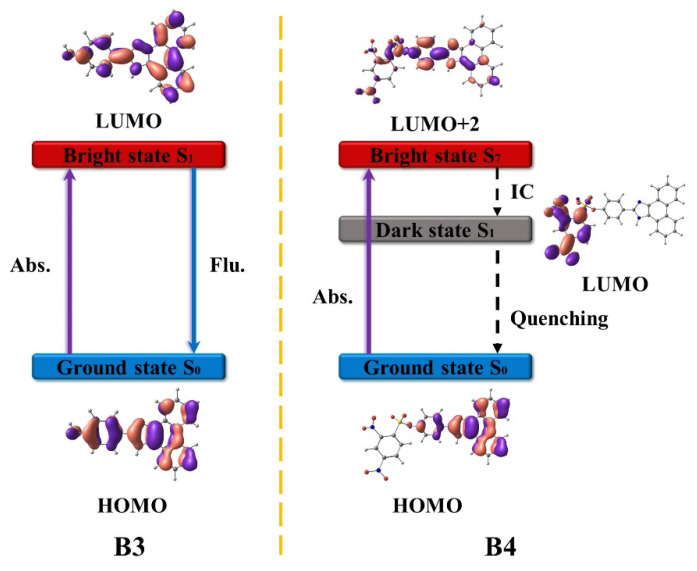
Scheme of the different sensing mechanisms and relevant frontier molecular orbitals B3 and B4.

We also calculated FMOs to better explain the sensing mechanism of B3 and B4 (Figure 5). Among them, the S_0_→S_1_ transition of B3 is mainly H→L, and the S_1_→S_0_ transition is mainly L→H, so we provide HOMO and LUMO. It can be seen that H→L is ππ * features, which provides evidence that the S_1_ state is a bright state. It is worth mentioning that we have not found the ICT process in H→L, but a local excitation (LE) process, which is inconsistent with the conclusion given by Wang et al. [28]. In order to further prove whether ICT process occurs in B3, we calculated the Δr index. The Δr index is a new method based on the hole-electron distance proposed by Guido et al. in 2013, which can be a good way to determine whether an ICT process is occurring or not [41]. The method has been widely accepted by researchers. Guido et al. suggested the use of 2.0 Å (Δr index) as a criterion to distinguish between LE and CT excitation. The Δr index value of B3 calculated by B3LYP functional is 1.13 Å, which can further indicate that B3 has no ICT process. Therefore, the sensing mechanism of B3 can be summarized: Firstly, B3 excitation from the S_0_ state to the S_1_ state, which is an LE process, followed by stabilization in the S_1_ state and finally back to the S_0_ state. Similarly, the S_0_→S_1_ and S_0_→S_7_ transition of B4 are mainly H→L and H→L+2, respectively, and the S_1_→S_0_ transition is mainly L→H, so we provide HOMO, LUMO, and LUMO+2. In Figure 5, the H→L+2 is ππ * features, also accompanied by the ICT process, which is similar to the A4 result. Meanwhile, the H→L shows a classic PET process. It is well known that the PET process is a non-radiative transition process, which is also the main reason for B4 fluorescence quenching. Therefore, the sensing mechanism of B4 can be summarized: Upon photo excitation, the B4 transition from the S_1_ state to the S_7_ state then undergoes an internal conversion to reach the S_1_ state, and finally undergoes a PET process from the S_1_ state back to the S_0_ state. Finally, because the B3LYP functional may underestimate the energy of CT state, we chose CAM−B3LYP functional to recalculate the excitation and emission energies and their related oscillator intensities, components, and FMOs (supporting information). The calculation results show that the trend is consistent with the results obtained by B3LYP functional calculation, further demonstrating the reliability and reasonableness of our chosen calculation method.

## 4. Conclusions

In summary, this work mainly investigates the fluorescence quenching mechanism of A4 and B4, and the fluorescence enhancement mechanism of A3 and B3. Compared to the A3 and B3 geometries, the A4 and B4 geometries are significantly more variable in the S_0_ and S_1_ states by geometric calculations, which is caused by the greater flexibility of the sulfonate groups on A4 and B4 than the hydroxyl groups on A3 and B3. Meanwhile, the calculated geometries and IR spectra demonstrate the enhanced intramolecular hydrogen bonding of A3−Enol, indicating susceptibility to the ESIPT processes. The PECs indicate that A3−Enol→A3−Keto is more likely to occur than A3−Keto→A3−Enol, and A3 Keto is more stable, which reasonably explains the enhanced fluorescence of ESIPT emission. The calculated electronic excitation energies, corresponding oscillator intensities, corresponding compositions, and FMOs well reveal that the fluorescence quenching of A4 and B4 is due to the PET mechanism. In addition, the S_1_ of B3 is a LE state and does not involve ICT characteristics, based on FMOs and Δr index. Our work not only elucidates that the fluorescence quenching pathways of A4 and B4 are caused by the PET process, but also demonstrates the cause of A3 and B3 fluorescence enhancement.

## Figures and Tables

**Table 1:** B3LYP-D3/TZVP/IEFPCM levels calculated electronic excitation energies (nm), corresponding oscillator intensities and corresponding compositions for the A3 and A4 compounds.

	Transition	λ (nm/eV)	*f* ^a^	Composition ^b^	CI (%) ^c^
A3	S_0_→S_1_	346/3.58	0.5677	H→L	81.51%
A4	S_0_→S_1_	664/1.87	0.0013	H→L	98.80%
	S_0_→S_2_	543/2.28	0.0000	H→L+1	98.77%
	S_0_→S_3_	489/2.54	0.0000	H-1→L	99.28%
	S_0_→S_4_	419/2.96	0.0000	H-1→L+1	99.18%
	S_0_→S_5_	393/3.16	0.0006	H-2→L	96.52%
	S_0_→S_6_	363/3.42	0.0047	H-3→L	78.51%
	S_0_→S_7_	351/3.53	0.6518	H→L+2	94.09%

^a^ Oscillator strength; ^b^ The main configurations are presented, in which H denotes the HOMO and, L stands for the LUMO; ^c^ The CI coefficients are in absolute values.

**Table 2:** B3LYP-D3/TZVP/IEFPCM levels calculated emission energies (nm), corresponding oscillator intensities and corresponding compositions for the A3 and A4 compounds.

	Transition	λ (nm/eV)	*f* ^a^	Composition ^b^	CI (%) ^c^
A3-Enol	S_1_→S_0_	415/2.99	1.2007	L→H	98.78%
A3-Keto	S_1_→S_0_	449/2.76	0.8211	L→H	98.90%
A4	S_1_→S_0_	1264/0.98	0.0059	L→H	99.47%

^a^ Oscillator strength; ^b^ The main configurations are presented, in which H denotes the HOMO and, L stands for the LUMO; ^c^ The CI coefficients are in absolute values.

**Table 3:** B3LYP−D3/TZVP/IEFPCM levels calculated electronic excitation energies (nm), corresponding oscillator intensities and corresponding compositions for the B3 and B4 compounds.

	Transition	λ (nm/eV)	*f* ^a^	Composition ^b^	CI(%) ^c^
B3	S_0_→S_1_	347/3.57	0.2500	H→L	70.13%
B4	S_0_→S_1_	681/1.82	0.0085	H→L	99.71%
	S_0_→S_2_	540/2.30	0.0003	H→L+1	99.78%
	S_0_→S_3_	487/2.55	0.0001	H-1→L	99.92%
	S_0_→S_4_	411/3.02	0.0000	H-1→L+1	99.94%
	S_0_→S_5_	393/3.16	0.0052	H-2→L	95.68%
	S_0_→S_6_	361/3.43	0.0011	H-3→L	67.93%
	S_0_→S_7_	358/3.46	0.5826	H→L+2	71.78%

^a^ Oscillator strength; ^b^ The main configurations are presented, in which H denotes the HOMO and, L stands for the LUMO; ^c^ The CI coefficients are in absolute values.

**Table 4:** B3LYP−D3/TZVP/IEFPCM levels calculated emission energies (nm), corresponding oscillator intensities and corresponding compositions for the B3 and B4 compounds.

	Transition	λ (nm/eV)	*f* ^a^	Composition ^b^	CI (%) ^c^
B3	S_1_→S_0_	442/2.81	1.3070	H→L	98.40%
B4	S_1_→S_0_	1272/0.97	0.0243	H→L	99.67%

^a^ Oscillator strength; ^b^ The main configurations are presented, in which H denotes the HOMO and, L stands for the LUMO; ^c^ The CI coefficients are in absolute values.

## Data Availability

The authors confirm that the data supporting the findings of this study are available within the article [and/or] its supplementary materials.

## Supplementary Materials

The following supporting information can be downloaded at: https://www.mdpi.com/article/10.3390/molecules27238444/s1, Figure S1: The frontier molecular orbitals of A3 and A4 forms in water solvent based on CAM−B3LYP−D3/TZVP/IEFPCM levels; Figure S2: The frontier molecular orbitals of B3 and B4 forms in DMF solvent based on CAM−B3LYP−D3/TZVP/IEFPCM levels; Table S1: CAM−B3LYP−D3/TZVP/IEFPCM levels calculated electronic excitation energies (nm), corresponding oscillator intensities and corresponding compositions for the A3 and A4 compounds; Table S2: CAM−B3LYP−D3/TZVP/IEFPCM levels calculated emission energies (nm), corresponding oscillator intensities and corresponding compositions for the A3 and A4 compounds; Table S3: CAM−B3LYP−D3/TZVP/IEFPCM levels calculated electronic excitation energies (nm), corresponding oscillator intensities and corresponding compositions for the B3 and B4 compounds; Table S4: CAM−B3LYP−D3/TZVP/IEFPCM levels calculated emission energies (nm), corresponding oscillator intensities and corresponding compositions for the B3 and B4 compounds.

## References

[1] Nunziata C., Polo A., Sorice A., Capone F., Accardo M., Guerriero E., Marino F.Z., Orditura M., Budillon A., Costantini S. (2019). Structural analysis of human SEPHS2 protein, a selenocysteine machinery component, over-expressed in triple negative breast cancer. Sci. Rep..

[2] Ge K., Xue A., Bai J., Wang S. (1983). Keshan disease-an endemic cardiomyopathy in China. Virchows Arch. A.

[3] Moreno-Reyes R., Suetens C., Mathieu F., Begaux F., Zhu D., Rivera M.T., Boelaert M., Neve J., Perlmutter N., Vanderpas J. (1998). Kashin-Beck osteoarthropathy in rural Tibet in relation to selenium and iodine status. N. Engl. J. Med..

[4] Rayman M.P. (2012). Selenium and human health. Lancet.

[5] Ercal N., Yang P., Aykin N. (2001). Determination of Biological Thiols by High-Performance Liquid Chromatography Following Derivatization by ThioGlo Maleimide Reagents. J. Chromatogr. B.

[6] Potesil D., Petrlova J., Adam V., Vacek J., Klejdus B., Zehnalek J., Trnkova L., Havel L., Kizek R. (2005). Simultaneous Femtomole Determination of Cysteine, Reduced and Oxidized Glutathione, and Phytochelatin in Maize (*Zea mays* L.) Kernels Using High-Performance Liquid Chromatography with Electrochemical Detection. J. Chromatogr. A.

[7] Ryant P., Dolezelova E., Fabrik I., Baloun J., Adam V., Babula P., Kizek R. (2008). Electrochemical Determination of Low Molecular Mass Thiols Content in Potatoes (*Solanum tuberosum*) Cultivated in the Presence of Various Sulphur Forms and Infected by Late Blight (*Phytophora infestans*). Sensors.

[8] Rafii M., Elango R., Courtney-Martin G., House J.D., Fisher L., Pencharz P.B. (2007). High-throughput and simultaneous measurement of homocysteine and cysteine in human plasma and urine by liquid chromatography–electrospray tandem mass spectrometry. Anal. Biochem..

[9] Burns E.A., Lawler E.A. (1963). Determination of mixtures of hydrazine and 1,1-dimethylhydrazine (UDMH) potentiometric and spectrophotometric end point detection. Anal. Chem..

[10] Rusin O., Luce N.N.S., Agbaria R.A., Escobedo J.O., Jiang S., Warner I.M., Dawan F.B., Lian K., Strongin R.M. (2004). Visual Detection of Cysteine and Homocysteine. J. Am. Chem. Soc..

[11] Sato Y., Iwata T., Tokutomi S., Kandori H. (2005). Reactive Cysteine is Protonated in the Triplet Excited State of the LOV2 Domain in *Adiantum* Phytochrome3. J. Am. Chem. Soc..

[12] Feng S., Fang Y., Feng W., Xia Q., Feng G. (2017). A colorimetric and ratiometric fluorescent probe with enhanced near-infrared fluorescence for selective detection of cysteine and its application in living cells. Dyes Pigment..

[13] Zhou P.W., Han K.L. (2018). Unraveling the detailed mechanism of excited-state proton transfer. Acc. Chem. Res..

[14] Zhao J.Z., Ji S.M., Chen Y.H., Guo H.M., Yang P. (2012). Excited state intramolecular proton transfer (ESIPT): From principal photophysics to the development of new chromophores and applications in fluorescent molecular probes and luminescentmaterials. Phys. Chem. Chem. Phys..

[15] Tang Z., Han H.Y., Ding J.X., Zhou P.W. (2021). Dual fluorescence of 2-(2′-hydroxyphenyl) benzoxazole derivatives via the branched decays from the upper excited-state. Phys. Chem. Chem. Phys..

[16] Kwon J.E., Park S.Y. (2011). Advanced organic optoelectronic materials: Harnessing excited state intramolecular proton transfer (ESIPT) process. Adv. Mater..

[17] Tang Z., Qi Y.T., Wang Y., Zhou P.W., Tian J., Fei X. (2018). Excited-state proton transfer mechanism of 2,6-diazaindoles·(H2O)n (n = 2–4) clusters. J. Phys. Chem. B.

[18] Chou P., McMorrow D., Aartsma T.J., Kasha M. (1984). The protontransfer laser. Gain spectra and amplification of spontaneous emission of 3-hydroxyflavone. J. Phys. Chem..

[19] Tang Z., Wei H.W., Zhou P.W. (2020). Effects of solvents on the excited state intramolecular proton transfer and hydrogen bond mechanisms of alizarin and its isomers. J. Mol. Liq..

[20] Tseng H.W., Liu J.Q., Chen Y.A., Chao C.M., Liu K.M., Chen C.L., Lin T.C., Hung C.H., Chou Y.L., Lin T.C. (2015). Harnessing excited-state Intramolecular proton transfer reaction via a series of amino-type hydrogen-bonding chromophore. J. Phys. Chem. Lett..

[21] Tang Z., Wang Y., Bao D., Lv M., Yang Y., Tian J., Dong L. (2017). Theoretical investigation of an excited-state intramolecular proton-transfer mechanism for an asymmetric structure of 3,7-dihydroxy-4-oxo-2-phenyl-4H-chromene-8-carbaldehyde: Single or double?. J. Phys. Chem. A.

[22] Hu R., Feng J., Hu D., Wang S., Li S., Li Y., Yang G. (2010). A Rapid Aqueous Fluoride Ion Sensor with Dual Output Modes. Angew. Chem. Int. Ed..

[23] Liu B., Wang H., Wang T., Bao Y., Du F., Tian J., Li Q., Bai R. (2012). A New Ratiometric ESIPT Sensor for Detection of Palladium Species in Aqueous Solution. Chem. Commun..

[24] Tang Z., Zhou P. (2020). New Insights into the Excited State Dynamics of Quinoline-Pyrazole Isomerism. J. Phys. Chem. B.

[25] Hanaoka K., Kikuchi K., Kojima H., Urano Y., Nagano T. (2004). Development of a Zinc Ion-Selective Luminescent Lanthanide Chemosensor for Biological Applications. J. Am. Chem. Soc..

[26] Zhao J., Jin B., Tang Z. (2022). Solvent-polarity-dependent conformation and ESIPT behaviors for 2-(benzimidazol-2-yl)-3-hydroxychromone: A novel dynamical mechanism. Phys. Chem. Chem. Phys..

[27] Ishikawa M., Sugisawa H., Fuchikami T., Kumada Y., Yamabe T., Kawakami H., Fukui K., Ueki Y., Shizuka H. (1982). Photolysis of Organopolysilanes. Photochemical Behavior of Phenylethynyldisilanes. J. Am. Chem. Soc..

[28] Wang Z., Su W., Zheng H., Yang S., Yang T., Han T., Dessie W., He X., Jiang Y., Hao Y. (2022). Two phenanthroimidazole turn-on probes for the rapid detection of selenocysteine and its application in living cells imaging. Spectrochim. Acta. A.

[29] Escudero D. (2016). Revising Intramolecular Photoinduced Electron Transfer (PET) from First-Principles. Acc. Chem. Res..

[30] Frisch M.J., Trucks G.W., Schlegel H.B., Scuseria G.E., Robb M.A., Cheeseman J.R., Scalmani G., Barone V., Mennucci B., Petersson G.A. (2016). Gaussian 16, Revision A.03.

[31] Becke A.D. (1993). Density-functional thermochemistry 0.3. The role of exact exchange. J. Chem. Phys..

[32] Lee C.T., Yang W.T., Parr R.G. (1988). Development of the Colle-Salvetti correlation-energy formula into a functional of the electron density. Phys. Phys. Rev. B.

[33] Miehlich B., Savin A., Stoll H., Preuss H. (1989). Results obtained with the correlation energy density functionals of Becke and Lee, Yang and Parr. Chem. Phys. Lett..

[34] Schafer A., Horn H., Ahlrichs R. (1992). Fully optimized contracted Gaussian basis sets for atoms Li to Kr. J. Chem. Phys..

[35] Grimmea S., Antony J., Ehrlich S., Krieg H. (2010). A consistent and accurate ab initio parametrization of density functional dispersion correction (DFT-D) for the 94 elements H-Pu. J. Chem. Phys..

[36] Mennucci B., Cances E., Tomasi J. (1997). Evaluation of Solvent Effects in Isotropic and Anisotropic Dielectrics and in Ionic Solutions with a Unified Integral Equation Method: Theoretical Bases, Computational Implementation, and Numerical Applications. J. Phys. Chem. B.

[37] Cances E., Mennucci B., Tomasi J. (1997). A new integral equation formalism for the polarizable continuum model: Theoretical background and applications to isotropic and anisotropic dielectrics. J. Chem. Phys..

[38] Miertus S., Scrocco E., Tomasi J. (1981). Electrostatic interaction of a solute with a continuum. A direct utilizaion of AB initio molecular potentials for the prevision of solvent effects. J. Chem. Phys..

[39] Cammi R., Tomasi J. (1995). Remarks on the use of the apparent surface charges (ASC) methods in solvation problems: Iterative versus matrix-inversion procedures and the renormalization of the apparent charges. J. Comput. Chem..

[40] Yanai T., Tew D.P., Handy N.C. (2004). A new hybrid exchangeecorrelation functional using the Coulomb-attenuating method (CAM-B3LYP). Chem. Phys. Lett..

[41] Guido C.A., Cortona P., Mennucci B., Adamo C. (2013). On the metric of charge transfer molecular excitations: A simple chemical descriptor. J. Chem. Theory. Comput..

