# Supplementary material for: Theoretical Investigations on the Sensing Mechanism of Phenanthroimidazole Fluorescent Probes for the Detection of Selenocysteine
Source: Molecules. 2022 Dec 2;27(23):8444. doi: 10.3390/molecules27238444 (PMC9735982; doi:10.3390/molecules27238444)
Supplement: Supplementary file 1 [file molecules-27-08444-s001.zip › molecules-2035259-supplementary.pdf]

Supporting information

Theoretical investigations on the sensing mechanism of phenanthroimidazole fluorescent probes for the detection of selenocysteine

Zhe Tang [a,b], Xiaochen Wang [b,c], Runze Liu [b]*, Panwang Zhou [b]*

[a] *Tianjin Key Laboratory of Drug Targeting and Bioimaging, Life and Health Intelligent Research Institute, Tianjin University of Technology, Tianjin 300384, China.*

[b] *Institute of Molecular Sciences and Engineering, Institute of Frontier and Interdisciplinary Science, Shandong University, Qingdao 266237, China.*

[c] *Key Laboratory of Materials Modification by Laser, Ion and Electron Beams, Ministry of Education, Dalian University of Technology, Dalian 116024, China*

Table S1. CAM-B3LYP-D3/TZVP/IEFPCM levels calculated electronic excitation energies (nm), corresponding oscillator intensities and corresponding compositions for the A3 and A4 compounds.

	Transition	λ(nm)	f [a]	Composition[b]	CI(%)[c]
A3	$S_0 \rightarrow S_1$	312	0.6783	H→L	82.96%
A4	$S_0 \rightarrow S_1$	353	0.0037	H→L	91.47%
	$S_0 \rightarrow S_2$	315	0.0001	H-11→L	71.65%
	$S_0 \rightarrow S_3$	312	0.0113	H-7→L+1	34.21%
	$S_0 \rightarrow S_4$	309	0.6359	H→L+2	74.14%

Table S2. CAM-B3LYP-D3/TZVP/IEFPCM levels calculated emission energies (nm), corresponding oscillator intensities and corresponding compositions for the A3 and A4 compounds.

	Transition	λ(nm)	f [a]	Composition[b]	CI(%)[c]
A3-Enol	$S_1 \rightarrow S_0$	378	1.2673	L→H	94.68%
A3-Keto	$S_1 \rightarrow S_0$	415	0.8502	L→H	91.75%
A4	$S_1 \rightarrow S_0$	499	0.0069	L→H	95.24%

Table S3. CAM-B3LYP-D3/TZVP/IEFPCM levels calculated electronic excitation energies (nm), corresponding oscillator intensities and corresponding compositions for the B3 and B4 compounds.

	Transition	λ(nm)	f [a]	Composition[b]	CI(%)[c]
B3	$S_0 \rightarrow S_1$	310	0.4681	H→L	77.54%
B4	$S_0 \rightarrow S_1$	346	0.0525	H→L	94.47%
	$S_0 \rightarrow S_2$	314	0.0000	H-11→L	67.23%
	$S_0 \rightarrow S_3$	312	0.8599	H→L+2	71.78%

Table S4. CAM-B3LYP-D3/TZVP/IEFPCM levels calculated emission energies (nm), corresponding oscillator intensities and corresponding compositions for the B3 and B4 compounds.

	Transition	λ(nm)	f [a]	Composition[b]	CI(%)[c]
B3	$S_1 \rightarrow S_0$	376	1.3738	H→L	95.74%
B4	$S_1 \rightarrow S_0$	476	0.0332	H→L	95.83%

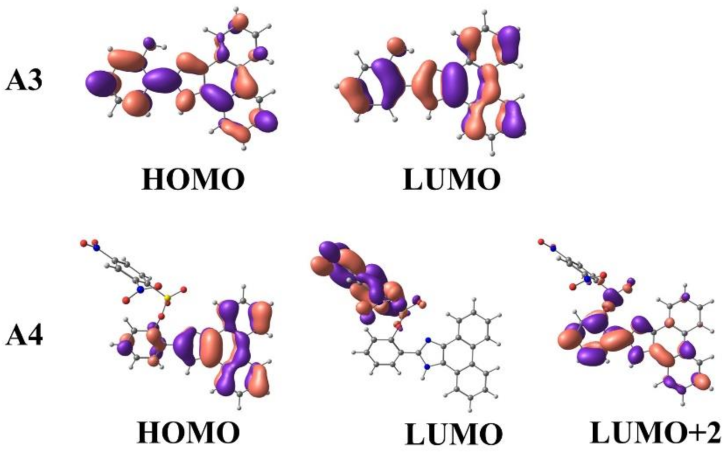

Figure S1. The frontier molecular orbitals of A3 and A4 forms in water solvent based on CAM-B3LYP-D3/TZVP/IEFPCM levels.

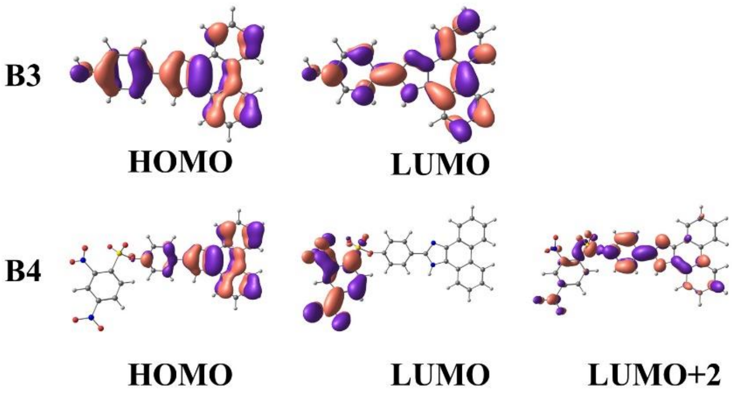

Figure S2. The frontier molecular orbitals of B3 and B4 forms in DMF solvent based on CAM-B3LYP-D3/TZVP/IEFPCM levels.

S_0 geometry of A3-Enol in Water solvent, B3LYP/TZVP/IEFPCM, empiricaldispersion=gd3

Energy=-993.818977

C	3.78897300	3.02368700	-0.00007700
C	3.91927000	1.64876900	0.00012500
C	2.79368100	0.79831100	0.00064000
C	1.51040500	1.41703900	-0.00074800
C	1.39364300	2.81878300	-0.00042800
C	2.51821000	3.61654300	-0.00049100
C	2.91412300	-0.66402700	-0.00095600
C	1.74878700	-1.48841200	0.00125500
C	0.49298700	-0.81551000	-0.00082700
C	0.36399000	0.56144400	0.00141700
C	4.16316000	-1.31784400	-0.00087700
C	4.26394200	-2.69531000	-0.00048100
C	3.10996300	-3.49076000	0.00071900
C	1.86864400	-2.89129800	0.00118800
N	-0.79530300	-1.30643000	-0.00070000
C	-1.64990100	-0.23662500	-0.00004700
N	-0.96690100	0.90507800	0.00095300
C	-3.10192100	-0.31028300	-0.00001600
C	-3.78857200	-1.53461100	-0.00050100
C	-5.17223500	-1.58665500	-0.00070000
C	-5.90424400	-0.39714400	-0.00042100
C	-5.25303900	0.82626800	0.00012000
C	-3.85747400	0.88926700	0.00026700
H	4.67573000	3.64521700	-0.00008900
H	4.91539800	1.22987800	0.00065600
H	0.40447300	3.25860400	-0.00071800
H	2.42160900	4.69512800	-0.00059300

H	5.07577400	-0.73917800	-0.00194500
H	5.24150800	-3.16082500	-0.00088000
H	3.19221500	-4.57028000	0.00110300
H	0.97754700	-3.50675300	0.00227500
H	-3.23314700	-2.46406600	-0.00063300
H	-5.67834000	-2.54292800	-0.00106800
H	-5.80706100	1.75633000	0.00041900
H	-1.06122600	-2.27761800	-0.00255800
H	-6.98679700	-0.42580300	-0.00053200
O	-3.28057900	2.11178300	0.00082800
H	-2.29197600	1.98770900	0.00122000

S_0 geometry of A3-Keto in Water solvent, B3LYP/TZVP/IEFPCM, empiricaldispersion=gd3

Energy=-993.808635

C	-3.78248800	3.02319200	-0.00144000
C	-3.91365600	1.64888900	-0.00050200
C	-2.78912500	0.79791600	0.00113400
C	-1.50630900	1.41970200	-0.00266700
C	-1.38726100	2.82186800	-0.00239600
C	-2.51244500	3.61735100	-0.00258400
C	-2.90812900	-0.66263100	-0.00189700
C	-1.74324500	-1.48722400	0.00204500
C	-0.48763300	-0.81572400	-0.00440000
C	-0.37530700	0.55473200	0.00304300
C	-4.15705300	-1.31710200	0.00021000
C	-4.25537100	-2.69402200	0.00142000
C	-3.10082500	-3.48902600	0.00257100
C	-1.85995200	-2.88995800	0.00203500
N	0.80522300	-1.32024200	-0.00355300
C	1.69239400	-0.29229800	-0.00112500

N	0.97363900	0.84729900	0.00148100
C	3.11941400	-0.32019400	-0.00097800
C	3.83345800	-1.53651600	-0.00385300
C	5.20996100	-1.55673100	-0.00324300
C	5.91066600	-0.33332100	0.00070000
C	5.24666600	0.87117500	0.00384800
C	3.81824500	0.95220600	0.00321500
H	-4.66890900	3.64482200	-0.00182600
H	-4.90946400	1.23002400	0.00017300
H	-0.40358300	3.27423900	-0.00374400
H	-2.41611400	4.69558300	-0.00332800
H	-5.07000800	-0.73935700	-0.00044700
H	-5.23225700	-3.16068400	0.00210400
H	-3.18273100	-4.56839400	0.00358000
H	-0.96928500	-3.50560900	0.00342000
H	3.29587300	-2.47810100	-0.00677100
H	5.74564900	-2.49663800	-0.00555200
H	5.79168800	1.80779400	0.00693600
H	1.05838000	-2.29485700	-0.00863800
H	6.99510100	-0.34285200	0.00127800
O	3.20766900	2.08128300	0.00638600
H	1.54025500	1.72066100	0.00540700

S_0 geometry of A4 in Water solvent, B3LYP/TZVP/IEFPCM, empiricaldispersion=gd3

Energy= -2182.725358

C	7.89909400	0.65707600	-0.42898500
C	7.13763000	-0.48325700	-0.26097500
C	5.73717000	-0.42679100	-0.11091700
C	5.13254000	0.86636700	-0.13699500
C	5.92278200	2.01869300	-0.30788500
C	7.29030800	1.91900500	-0.45282700

C	4.91854700	-1.63289800	0.06790500
C	3.50667200	-1.52944600	0.21809900
C	2.93225800	-0.21836400	0.20156900
C	3.71587700	0.90954100	0.01837300
C	5.47535400	-2.92895500	0.08919600
C	4.68743400	-4.05247000	0.24832700
C	3.29725000	-3.93178700	0.38874000
C	2.71474400	-2.68167900	0.37347300
N	1.61188400	0.12902600	0.32968300
C	1.57472900	1.44451000	0.22908400
N	2.83630700	1.96564800	0.04366600
C	0.38195700	2.28274900	0.32523700
C	-0.89452800	1.81412700	-0.01877100
C	-2.01805400	2.62316700	0.04362800
C	-1.89713300	3.93912800	0.47290300
C	-0.64557600	4.43154300	0.83093000
C	0.47377900	3.61461700	0.75646300
O	-1.04958900	0.51962700	-0.53993500
S	-1.41173000	-0.73395200	0.49855900
O	-0.85839200	-1.94302500	-0.10130200
O	-1.12163600	-0.34999800	1.88065800
C	-3.23089400	-0.74236400	0.33412300
C	-3.95005100	-0.94418700	1.50488700
C	-5.33913500	-0.93825100	1.48445500
C	-5.98515800	-0.70097600	0.28343500
C	-5.29716300	-0.46811600	-0.89679900
C	-3.91685600	-0.51619200	-0.86003900
N	-3.22387200	-0.29475100	-2.15252700
O	-3.52140200	0.71848700	-2.76615300
O	-2.43701900	-1.14746600	-2.52328900

N	-7.46843200	-0.67550700	0.25156300
O	-8.01006000	-0.47557800	-0.82655800
O	-8.06101800	-0.85716300	1.30504200
H	8.97268800	0.57244700	-0.54149900
H	7.64407900	-1.43780100	-0.24598600
H	5.45219200	2.99406000	-0.32270700
H	7.88981900	2.81103500	-0.58334400
H	6.54191000	-3.06382200	-0.02171400
H	5.14919400	-5.03201000	0.26010000
H	2.68485500	-4.81726800	0.50687100
H	1.64246000	-2.56284100	0.46774000
H	3.05988800	2.93349200	-0.12551400
H	-2.97712500	2.22289400	-0.25497300
H	-2.77342100	4.57149000	0.52413000
H	-0.54018300	5.45237800	1.17403400
H	1.43384300	4.00930200	1.06209300
H	-3.42663100	-1.08225600	2.43991600
H	-5.90545500	-1.10214200	2.38876700
H	-5.82017300	-0.26446500	-1.81908700

S_0 geometry of B3 in Water solvent, B3LYP/TZVP/IEFPCM, empiricaldispersion=gd3

Energy=-993.809751

C	4.18420000	2.92191000	-0.06112400
C	4.22825000	1.54197000	-0.02808300
C	3.05196900	0.76268200	-0.01534700
C	1.80812100	1.45799700	-0.03629600
C	1.78059800	2.86429200	-0.07066700
C	2.95212100	3.59136300	-0.08237100
C	3.08357700	-0.70365800	0.02277800
C	1.86929800	-1.45456400	0.02966900
C	0.65755600	-0.70595700	0.01220300

C	0.60726100	0.67725300	-0.02622300
C	4.29041100	-1.43222200	0.04711800
C	4.30773200	-2.81301600	0.07750100
C	3.10703800	-3.53626500	0.08329900
C	1.90463900	-2.86237100	0.06039100
N	-0.65767500	-1.11190900	0.01311600
C	-1.44121200	0.01770400	-0.01567900
N	-0.69741500	1.11238700	-0.04117300
C	-2.90157400	-0.01293400	-0.01274700
C	-3.62580200	-1.17977000	-0.29572700
C	-5.01162400	-1.18250100	-0.28274000
C	-5.70762600	-0.00941400	0.00895200
C	-5.00296300	1.16413900	0.28569900
C	-3.61716600	1.15676700	0.27517800
O	-7.07120200	-0.07142300	0.00661700
H	5.10788500	3.48718200	-0.07056200
H	5.19654400	1.06214600	-0.01307500
H	0.81986800	3.36247500	-0.08747800
H	2.92209900	4.67367700	-0.10834300
H	5.23656200	-0.91000300	0.04335600
H	5.25513900	-3.33697000	0.09609500
H	3.12311100	-4.61866000	0.10582600
H	0.97783400	-3.42267700	0.06342800
H	-3.11595100	-2.10077800	-0.54883700
H	-5.56587000	-2.08490600	-0.50545500
H	-5.53946400	2.07773400	0.51418500
H	-3.07444000	2.06584700	0.49583700
H	-7.44891600	0.79267300	0.21559900
H	-0.98797800	-2.06013900	0.08818200

S_0 geometry of B4 in Water solvent, B3LYP/TZVP/IEFPCM, empiricaldispersion=gd3

Energy=-2182.723152

C	-8.60346000	2.16187800	-0.96937600
C	-8.42892300	0.81003000	-0.74628900
C	-7.16705700	0.27003400	-0.42018700
C	-6.07266300	1.17781500	-0.32995900
C	-6.26675600	2.55164900	-0.55983900
C	-7.51618800	3.04215300	-0.87619500
C	-6.96633400	-1.16454300	-0.18190300
C	-5.67300100	-1.67124700	0.14453100
C	-4.61676400	-0.71789100	0.22014600
C	-4.78634600	0.63963000	0.00014800
C	-8.02014200	-2.09761200	-0.26025300
C	-7.81801400	-3.44524300	-0.03373100
C	-6.54109200	-3.92720100	0.28461300
C	-5.48310500	-3.04763000	0.37170700
N	-3.28185100	-0.87079800	0.50876000
C	-2.70060000	0.37292900	0.44644300
N	-3.59109800	1.30224400	0.14089400
C	-1.27865700	0.61329300	0.68343300
C	-0.45490700	-0.34754400	1.28372700
C	0.89603400	-0.09903500	1.47880900
C	1.42418200	1.12071500	1.08099600
C	0.62545600	2.10890900	0.51899900
C	-0.71936200	1.84418100	0.31322300
O	2.77932800	1.34973100	1.38268500
H	-9.58705100	2.54082400	-1.21775800
H	-9.29099100	0.16338600	-0.82801100
H	-5.41459000	3.21456100	-0.48309500
H	-7.65778800	4.10133400	-1.05204700

H	-9.01923700	-1.76505300	-0.50292000
H	-8.65297700	-4.13111300	-0.10309900
H	-6.38582300	-4.98399800	0.46137800
H	-4.49606300	-3.41920800	0.61811600
H	-0.85595100	-1.29328600	1.62339300
H	1.52883500	-0.83290000	1.95913400
H	1.04812200	3.06286500	0.24073300
H	-1.35199400	2.59713700	-0.13546100
H	-2.80646300	-1.74263000	0.67710600
S	3.89293500	1.83561900	0.24704100
O	4.76897100	2.73763500	0.99146000
O	3.20411600	2.24479800	-0.97731000
C	4.80013800	0.28122300	-0.05443500
C	4.10771800	-0.91885500	0.01150200
C	6.17242700	0.26757000	-0.31405000
C	4.77659400	-2.12457500	-0.15851400
H	3.04837600	-0.93123700	0.21020200
C	6.86275200	-0.92092500	-0.45342400
C	6.14286100	-2.10216500	-0.37760600
H	4.24295700	-3.06169100	-0.11043500
H	7.92675000	-0.92294900	-0.63645200
N	6.95686700	1.50759300	-0.52254000
N	6.87167800	-3.38459500	-0.54025700
O	8.03813400	1.58659500	0.03577000
O	6.48218600	2.34109500	-1.27840400
O	8.07857800	-3.33177500	-0.72886500
O	6.22106100	-4.41711900	-0.47490500

S_1 geometry of A3-Enol in Water solvent, B3LYP/TZVP/IEFPCM, empiricaldispersion=gd3

Energy=-993.700289

C	-3.78022200	3.03019500	-0.00047800
C	-3.91362200	1.64939700	0.00100900
C	-2.79482700	0.79734900	-0.00240200
C	-1.50960500	1.42283100	-0.00097900
C	-1.39106300	2.82231700	-0.00404200
C	-2.51535900	3.62776500	-0.00256700
C	-2.91478800	-0.66065600	0.00659300
C	-1.72997400	-1.49025000	-0.00587200
C	-0.49130700	-0.84050900	0.00390200
C	-0.35037400	0.58255500	-0.01240000
C	-4.15070500	-1.31904900	0.01193600
C	-4.24870400	-2.70396000	0.01036500
C	-3.08975600	-3.50414000	-0.00153000
C	-1.85340600	-2.90756600	-0.00848400
N	0.78680600	-1.32784600	-0.00295800
C	1.66995100	-0.23477500	-0.00345400
N	0.94043500	0.92923300	-0.01382200
C	3.06284600	-0.30922400	-0.00504100
C	3.77580000	-1.55809700	-0.01286800
C	5.15238900	-1.58861900	-0.00477400
C	5.89913200	-0.39710700	0.00949200
C	5.24180700	0.83967000	0.01327500
C	3.85783500	0.90699400	0.00549500
H	-4.66965600	3.64788600	0.00060700
H	-4.91175100	1.23552500	0.00263200
H	-0.40064800	3.25909800	-0.00583700
H	-2.41807400	4.70552900	-0.00379600
H	-5.06818500	-0.74748700	0.01928000
H	-5.22607100	-3.16931300	0.01613200
H	-3.17451500	-4.58324700	-0.00568600

H	-0.96139800	-3.52132800	-0.02158400
H	3.22475600	-2.48919000	-0.03026600
H	5.66542500	-2.54266800	-0.01139600
H	5.79783600	1.76881500	0.02150400
H	1.05310800	-2.29542700	0.06594900
H	6.98094200	-0.43198700	0.01590000
O	3.27233800	2.12085600	0.00897400
H	2.28255200	1.99650200	-0.00083300

S_1 geometry of A3-Keto in Water solvent, B3LYP/TZVP/IEFPCM, empiricaldispersion=gd3

Energy=-993.701522

C	-3.87297900	2.97470600	-0.00601400
C	-3.97158300	1.59738800	-0.00359400
C	-2.82969700	0.76938500	-0.00393800
C	-1.55744500	1.42605900	-0.00153300
C	-1.47504700	2.83228400	-0.00527500
C	-2.61696900	3.60300500	-0.00662500
C	-2.91370000	-0.68576200	0.00410600
C	-1.71892400	-1.48376900	-0.00070000
C	-0.48232000	-0.79621800	0.00512000
C	-0.39936800	0.59755800	-0.00705900
C	-4.14166600	-1.37562000	0.00553400
C	-4.20499200	-2.75584400	0.00681700
C	-3.02638500	-3.52453200	0.00394300
C	-1.80398800	-2.89496900	0.00101400
N	0.80690700	-1.27038900	0.00386100
C	1.70577600	-0.21108300	0.00312400
N	0.92057300	0.92294600	-0.00626100
C	3.11316000	-0.28104700	-0.00279600
C	3.80224600	-1.50884100	-0.01965200

C	5.20231100	-1.55503800	-0.01659100
C	5.96353100	-0.38072400	-0.00136300
C	5.32455600	0.85796700	0.01080600
C	3.90505100	0.97419600	0.01071200
H	-4.77499700	3.57373800	-0.00689800
H	-4.95789600	1.15623200	-0.00310100
H	-0.50441200	3.31266000	-0.00576600
H	-2.54662200	4.68297400	-0.00854400
H	-5.07069900	-0.82326800	0.00706000
H	-5.16971000	-3.24715500	0.00899400
H	-3.08180300	-4.60561300	0.00387000
H	-0.89817300	-3.48849700	-0.00295000
H	3.25645000	-2.44371400	-0.03679500
H	5.69599900	-2.51879800	-0.02797800
H	5.88852500	1.78282000	0.02129200
H	1.07340900	-2.23973800	0.03872400
H	7.04490100	-0.43640200	0.00029200
O	3.31994200	2.10299600	0.02227600
H	1.38370500	1.82827700	-0.00580900

S_1 geometry of A4 in Water solvent, B3LYP/TZVP/IEFPCM, empiricaldispersion=gd3

Energy=-2182.673536

C	-7.89215800	0.92870000	-0.02906300
C	-7.16651900	-0.25542400	-0.07355400
C	-5.76894800	-0.25676600	-0.06178200
C	-5.12150500	1.01843300	-0.01164800
C	-5.87187800	2.21224200	0.03579200
C	-7.24827000	2.16855600	0.02622500
C	-4.98366600	-1.50783000	-0.11146900
C	-3.56140000	-1.47080700	-0.09294400
C	-2.93687600	-0.19632200	-0.05147700

C	-3.71028300	1.00668900	0.00045900
C	-5.58524000	-2.76873800	-0.16079000
C	-4.82202200	-3.93247000	-0.19646800
C	-3.42721200	-3.87933600	-0.17925600
C	-2.79624900	-2.65137700	-0.12819200
N	-1.62544400	0.08925900	-0.02679500
C	-1.53099100	1.41543500	0.02617200
N	-2.79495700	2.00070500	0.03920800
C	-0.31059200	2.18532400	0.04814100
C	0.93671300	1.59698300	0.36462700
C	2.09152700	2.35966900	0.40668600
C	2.04223900	3.71829300	0.11241100
C	0.82793200	4.31926300	-0.21417900
C	-0.33041400	3.56461000	-0.23767300
O	1.01101400	0.26397700	0.72806200
S	1.39447700	-0.87154400	-0.48455600
O	0.80084900	-2.12302900	-0.01269500
O	0.97751000	-0.32069300	-1.78284900
C	3.17133100	-0.83216000	-0.42674400
C	3.82216100	-0.72166500	-1.66728600
C	5.18212600	-0.54170000	-1.75638700
C	5.94814600	-0.43374700	-0.57340700
C	5.31980000	-0.51757100	0.67324800
C	3.96308800	-0.73561000	0.75052800
N	3.42520100	-0.89566900	2.09787100
O	3.93590400	-0.23817100	3.00936600
O	2.50150900	-1.69101300	2.26208100
N	7.33905400	-0.23879500	-0.63189700
O	8.00128600	-0.14127000	0.44407700
O	7.88976000	-0.15756600	-1.76858500

H	-8.97335200	0.88500700	-0.03831500
H	-7.71418700	-1.18487500	-0.11604100
H	-5.36385300	3.16650500	0.07658800
H	-7.82480700	3.08270700	0.06101700
H	-6.66049600	-2.86626500	-0.17284000
H	-5.32460200	-4.89013700	-0.23578800
H	-2.84652900	-4.79166200	-0.20520500
H	-1.71637700	-2.57159600	-0.10887100
H	-2.99295900	2.98536200	0.13217300
H	3.02458700	1.88496800	0.67435800
H	2.95065700	4.30512700	0.14243100
H	0.78686300	5.37386500	-0.44973100
H	-1.25757500	4.04896800	-0.51165200
H	3.23294000	-0.75632300	-2.57182200
H	5.66888300	-0.46875400	-2.71543800
H	5.90451000	-0.44062300	1.57582300

S_1 geometry of B3 in Water solvent, B3LYP/TZVP/IEFPCM, empiricaldispersion=gd3

Energy=-993.689813

C	-4.15653700	2.94593800	0.00431000
C	-4.21302400	1.56003900	0.00329000
C	-3.04707800	0.76806600	0.00333800
C	-1.79766200	1.46603700	0.00021600
C	-1.75897200	2.86657900	0.00223500
C	-2.92760600	3.61279900	0.00308300
C	-3.08678100	-0.69059700	-0.00577700
C	-1.85588200	-1.45986400	0.00414000
C	-0.65546700	-0.74287200	-0.00462900
C	-0.58667000	0.68896000	0.00973700
C	-4.28551400	-1.41835400	-0.01212700
C	-4.30982100	-2.80501200	-0.01185700

C	-3.10715800	-3.54469000	-0.00148100
C	-1.90585000	-2.88157700	0.00520400
N	0.64740300	-1.15292700	0.00188600
C	1.46489000	-0.00480000	0.00058400
N	0.67697700	1.11467200	0.00936000
C	2.86503300	-0.02564000	0.00408300
C	3.62330300	-1.24493900	0.02060400
C	4.99868700	-1.22312200	0.01634200
C	5.69252100	-0.00270900	-0.00390900
C	4.97633300	1.21003200	-0.01703900
C	3.60308200	1.20465800	-0.01251600
O	7.05628700	-0.05483600	-0.00765600
H	-5.07955700	3.51281700	0.00565900
H	-5.18699500	1.09162200	0.00459900
H	-0.79334800	3.35577500	0.00200800
H	-2.88869000	4.69433100	0.00380600
H	-5.23290500	-0.89724500	-0.01896800
H	-5.26109200	-3.32197200	-0.01793700
H	-3.13416500	-4.62681000	0.00149000
H	-0.98216600	-3.44715800	0.01634100
H	3.12402400	-2.20468600	0.04396200
H	5.56581800	-2.14549100	0.03051900
H	5.51548200	2.15081800	-0.03072800
H	3.05733700	2.13772200	-0.02303900
H	7.42830800	0.83706400	-0.01801300
H	0.96802700	-2.10390200	-0.06415500

S_1 geometry of B4 in Water solvent, B3LYP/TZVP/IEFPCM, empiricaldispersion=gd3

Energy=-2182.672284

C	8.42603500	-2.34228700	-1.24237200
C	8.36914800	-0.99336700	-0.91242500

C	7.16901200	-0.38979100	-0.51907400
C	6.01061200	-1.21497700	-0.47713700
C	6.08000000	-2.57832900	-0.81048300
C	7.28177400	-3.14074600	-1.19374000
C	7.08628500	1.04398200	-0.17466600
C	5.84899800	1.62944700	0.23892500
C	4.72716400	0.77411200	0.26635300
C	4.78500600	-0.61589200	-0.06409800
C	8.20058100	1.88719200	-0.22080600
C	8.10808400	3.23229600	0.11330800
C	6.89131600	3.79446300	0.51220900
C	5.76937600	2.99833600	0.57220600
N	3.43336600	0.99699100	0.59420200
C	2.74878800	-0.19925300	0.45942100
N	3.57089000	-1.17646300	0.05922800
C	1.34388800	-0.35973800	0.71022800
C	0.52832000	0.71116800	1.12439300
C	-0.81976100	0.51690300	1.33840500
C	-1.37513300	-0.74961600	1.14085700
C	-0.58166200	-1.83462500	0.76110700
C	0.76495000	-1.63377200	0.53947900
O	-2.71445300	-0.87042800	1.43903500
H	9.37285200	-2.77342000	-1.54061700
H	9.28200900	-0.41896100	-0.96487200
H	5.17734800	-3.17233900	-0.76247700
H	7.33660600	-4.18973900	-1.45230400
H	9.16351100	1.50464800	-0.52379000
H	8.99464700	3.85070900	0.06280900
H	6.83193700	4.84315700	0.76927300
H	4.82339300	3.42464000	0.87884300

H	0.93375700	1.70141100	1.27827000
H	-1.45324000	1.33054000	1.66241300
H	-1.01787300	-2.81391000	0.64138000
H	1.39207700	-2.46112300	0.24020500
H	3.03996300	1.87874300	0.88528800
S	-3.84541900	-1.68377800	0.45247400
O	-4.56934400	-2.52971900	1.40367900
O	-3.10452300	-2.27593200	-0.66808900
C	-4.84113900	-0.29437300	-0.04026200
C	-4.24946000	0.97141700	-0.14664100
C	-6.23901900	-0.39997400	-0.25766000
C	-5.00159100	2.09401000	-0.40735800
H	-3.18706400	1.08310800	-0.00095600
C	-7.00729900	0.71669500	-0.48863100
C	-6.40113200	1.97713800	-0.56027700
H	-4.53628800	3.06305000	-0.48864800
H	-8.06644200	0.61726500	-0.66498900
N	-6.91537200	-1.68652400	-0.39529000
N	-7.19213700	3.10883200	-0.81817200
O	-8.09803400	-1.76983600	-0.06525500
O	-6.26564400	-2.62137500	-0.86831900
O	-8.44808000	2.97645500	-0.94313100
O	-6.62199000	4.23712200	-0.91126500
